# Ultrafast Synthesis of Multifunctional Submicrometer Hollow Silica Spheres in Microfluidic Spiral Channels

**DOI:** 10.1038/s41598-017-12856-9

**Published:** 2017-10-03

**Authors:** Yuan Nie, Nanjing Hao, John X. J. Zhang

**Affiliations:** Thayer School of Engineering, Dartmouth College, 14 Engineering Drive, Hanover, New Hampshire 03755 United States

## Abstract

We demonstrate a facile and ultrafast approach for the synthesis of multifunctional submicrometer hollow silica spheres (smHSSs) using microfluidic spiral channels with enhanced mixing performance, introduced by the transverse Dean flows cross the channel as a result of centrifugal effects. Formation of smHSSs is initiated by the hydrolysis of tetraethyl orthosilicate (TEOS) at the interface of two laminar reactant flows. Complete mixing of the flows further facilitates the subsequent condensation of hydrolyzed TEOS, which builds up the shell layer of smHSSs. The average size of the as-synthesized smHSSs is 804.7 nm, and the thickness of the shell layer is ~20 nm. Multifunctional smHSSs integrated with proteins, fluorescent dyes, quantum dots, and magnetic nanoparticles can be further produced via this general platform. Their applications in cell imaging, organic dye adsorption, and drug delivery are examined.

## Introduction

Hollow silica materials have given rise to great interest due to their unique properties such as low density, large void space, high surface-to-volume ratio, excellent loading capacity and good biocompatibility^1–3^, which endow them with great opportunities in adsorption and separation, controlled drug delivery, and biosensing^4–6^. Conventional batch procedures for the fabrication of hollow silica materials include hard templates^7,8^, layer-by-layer (LBL) assembly^9^, and emulsions with oil-water interface^10,11^. Hard templates method involves multi-step operations including preparation of hard templates, functionalization and coating of the template surface and selective removal of the templates. LBL assembly technique requires a sequential deposition of oppositely charged polymer molecules via electrostatic interactions. Both methods are time consuming, low-yield-rate, hard to scale up and to be reproducible. The emulsions method is relatively simple as it requires only mixing of two immiscible liquids by shaking or stirring. However, these emulsions are not stable and the size of the synthesized hollow structures is hard to control.

Compared with those macroscale based methods, microfluidics offers many benefits in efficient and reproducible synthesis of micro and nanostructures with excellent control of their physicochemical properties^12^. Down to microscale, it requires very low consumption of reagents and generates relatively high mass transport rates. The combination of the two advantages enables more aggressive reactions rates, which result in considerably high product yields. However, only a few studies have been reported in microfluidics-based flow synthesis of silica materials. Most of the synthesized particles are solid structures with diameters of several micrometers^13,14^. There are also some large-sized hollow silica microspheres with diameters of tens or hundreds of micrometer scales^15^. Silica nanospheres with particle sizes of submicrometers were reported^16–20^, but these nanospheres were mainly with solid structures fabricated at relatively low flow rates. Therefore, the synthesis of hollow silica nanospheres in a simple and high-throughput way remains a field unexplored to some extent. Moreover, existing microreactors for the synthesis of silica materials mainly focused on droplet generation with different geometries such as T-shaped device and cross-channel device^21,22^. In these microreactors, droplets are usually created by two immiscible fluids: tetraethyl orthosilicate (TEOS) as silica precursor in oil phase and catalyst in water phase. The formation of silica particles relies on interfacial polymerization (hydrolysis and condensation of TEOS), which results in diffusion and reaction limited performances confined by the droplets. Till now, limited work has been done in improving mixing performance in microfluidics-based flow synthesis of hollow silica materials. Even less work has been reported on the synthesis of multifunctional silica materials. Therefore, developing new mixing-enhanced microfluidic devices for the synthesis of hollow silica spheres, especially multifunctional ones integrated with other materials, is still a big challenge and in great demand.

Herein, we demonstrate the synthesis of hollow silica spheres at submicrometer scale in a microfluidic spiral channel, which combines diffusion-limited reactions at the interface of two laminar flows near the inlets and therefore does not require the generation of droplets, and enhanced instant mixing as the interfacial area of two miscible phases is stretched and extended across the microchannel because of transverse Dean flow effects. The flow rates are tunable, which makes the synthesis process much more flexible. By increasing the flow rate to 400 μL/min, submicrometer hollow silica spheres (smHSSs) could be synthesized in less than one second. Compared with batch mode methods which usually take hours for fabricating the silica spheres, the microfluidic synthesis method we propose here is ultrafast. The synthesis mechanism has been experimentally investigated. Furthermore, the simplicity and versatility of this method largely facilitate the fabrication of multifunctional smHSSs loaded with proteins, fluorescent dyes, quantum dots, and magnetic nanoparticles for diverse applications such as cell imaging, dye adsorption, and drug delivery.

## Results

### Design and fabrication of microfluidic spiral channel

For the formation of smHSSs, firstly an interface of silica precursor in non-aqueous phase and catalyst in aqueous phase is needed where the hydrolysis of TEOS occurs, then continuous condensation of hydrolyzed TEOS with mixing of the two reactant flows builds up the shell layer. For this purpose, we chose microfluidic spiral channel as: (1) with low Reynolds number (*Re* < ~2000), viscous-dominated laminar flows are common in microfluidic devices, which facilitates creating the interface of two reactant flows; and (2) in microfluidic spiral channels, mixing is largely enhanced with the introduction of chaotic advection due to the transverse Dean flow across the channel as a result of centrifugal effects^23^.

The magnitude of the Dean flow effects is described by a dimensionless Dean number as *κ* = (*D*_*H*_/(2R))^1/2^ *Re*, where *D*_*H*_ is microchannel hydraulic diameter, R is the flow path radius of curvature and *Re* is the Reynolds number^24^. The five-run spiral microchannel has two inlets for the reactants and one outlet for products. The width and height are 500 μm and 50 μm, respectively. The largest synthesis flow rate we used is 400 μL/min. In this case, the average velocity in the microchannel is *U*_*ave*_ = (flow rate)/(cross section area) = 0.27 m/s. And the Reynolds number is *Re* = $\rho \,$ *U*_*ave*_ *D*_*H*_/*μ* = 24.5, where the density (~1000 kg/m^3^) and dynamic viscosity (~0.001 Pa·s) of water are used for approximations. The smallest diameter of the microchannel is 5.25 mm, then it increases from 11.0 mm to 22.2 mm with an increment of 1.4 mm for each half run. For the innermost part, the Dean number is calculated to be $\kappa \,=$ 2.28.

**Figure 1:**
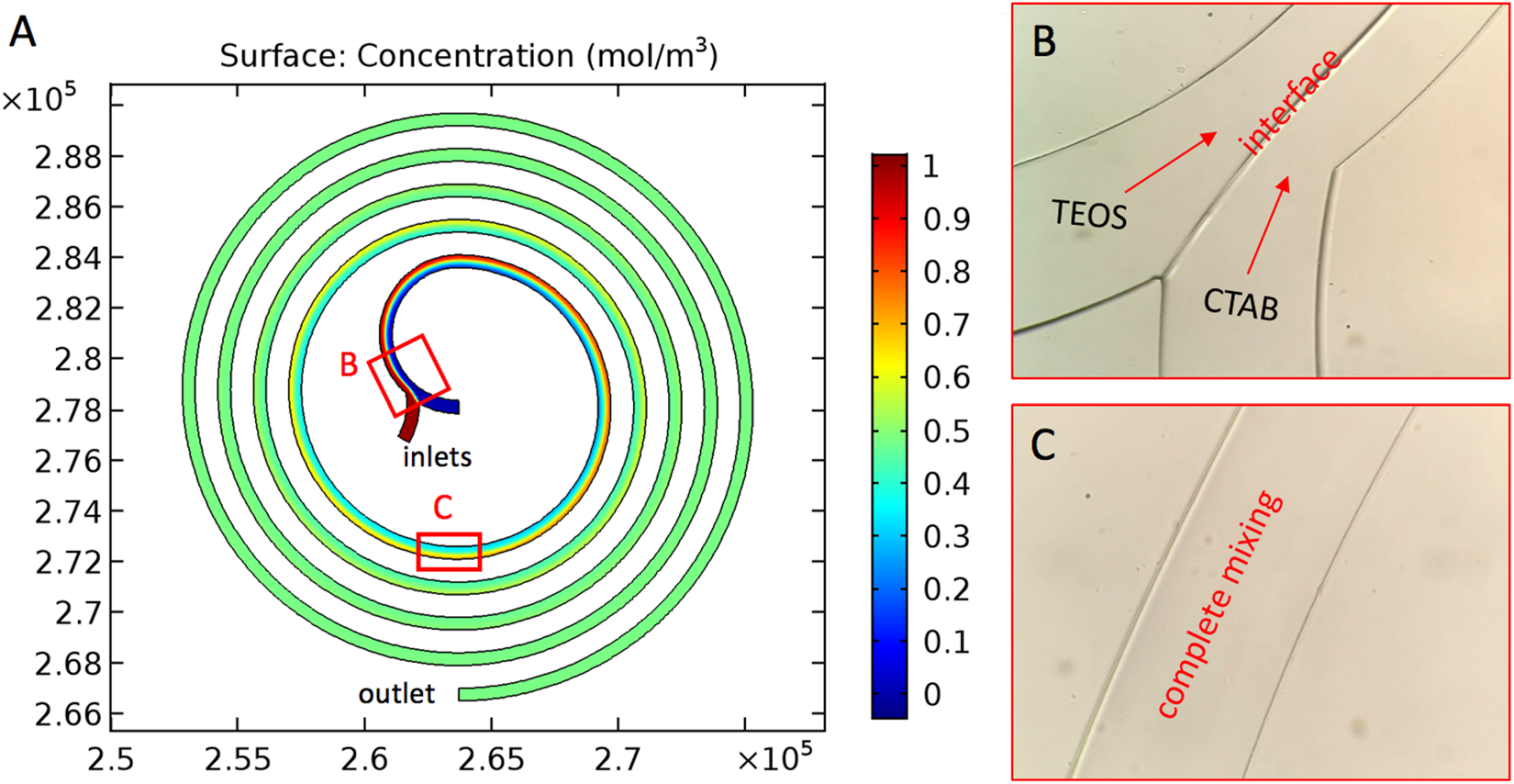
Simulation and experimental results of mixing in spiral microchannel. (**A**) two flows with different concentrations mix in spiral channel: by simulation, a clear interface of two fluids is predicted near the inlets, and complete mixing is achieved after about one run; Experimental observation is consistent with simulation prediction: (**B**) the interface of two reaction flows, and (**C**) complete mixing after one run of the spiral channel.

We simulated mixing in spiral microchannels as shown in Fig. 1A. The Reynolds number calculated was 24.5 (<2300), and laminar flows were expected in the microchannel. The material properties were those of water (density ~1000 kg/m^3^ and dynamic viscosity ~0.001 Pa·s). A diffusion coefficient $D=5\times {10}^{-10}\,{m}^{2}/s$ was used for the fluids^25,26^. Fluid flow rate was 400 μL/min. Complete mesh for simulations consisted of 14,035 elements. We considered the flows to be incompressible with no-slip boundary condition and neglected the gravity force. The outlet was set to a fixed-pressure (*p*) boundary condition (*p* = *0*).

Then we fabricated the spiral microchannels using soft lithography and tested their mixing performance. In Fig. 1B and C, the interface of two laminar flows near the inlets and complete mixing of the fluids after around one run of the spiral microchannel were clearly observed, demonstrating consistency of simulation and experimental results. The short diffusion length proved an enhancement of mixing in spiral microchannels.

### Synthesis of smHSSs in spiral microchannel

To fabricate smHSSs with uniform size in a fast way, a series of reacting parameters including chemical compositions, concentrations and flow rates were carefully investigated. We chose TEOS (80 μL) in pure ethanol (30 mL) as one reactant flow and ammonium hydroxide (1.5 mL) and hexadecyltrimethylammonium bromide (CTAB) (160 mg) in deionized (DI) water (30 mL) as the other. The two flows were pumped (PHD-2000, Harvard Apparatus) into the spiral microchannel through the inlets and the as-synthesized products were collected at the outlet. The flow rate we used was 400 μL/min. To estimate the time for synthesis, firstly, the length of the channel is calculated, which is 25.07 cm. And the volume of fluids in the channel is 6.27 μL. The time for the synthesis of smHSSs is t = (volume)/(flow rate) = 0.94 s, which is less than one second. Therefore, the method we proposed here to fabricate hollow silica nanomaterials is ultrafast compared to batch mode techniques.

### Characterization of smHSSs

**Figure 2:**
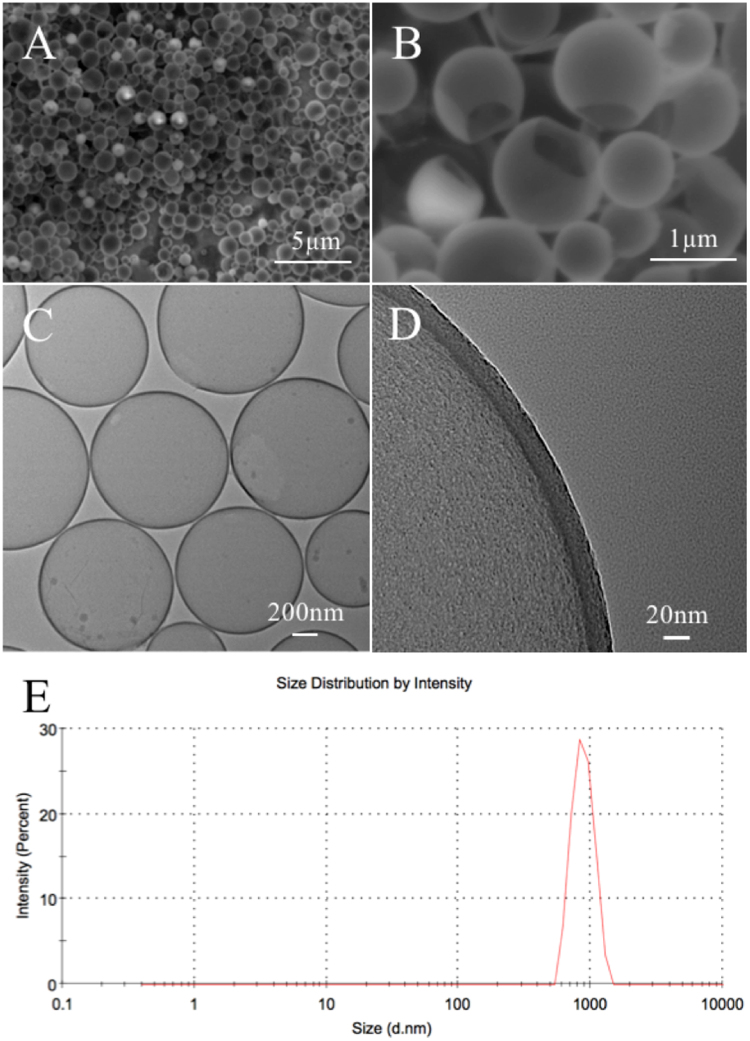
SEM (**A**,**B**) and TEM (**C**,**D**) images of as-synthesized smHSSs, (**E**) Dynamic Light Scattering (DLS) size distribution analysis of the smHSSs.

The smHSSs were characterized using SEM (Fig. 2A,B) and TEM (Fig. 2C,D), and a statistical size distribution (Fig. S1) was obtained by analyzing TEM images of 300 spheres using ImageJ software. The average diameter was calculated to be 804.7 nm and the standard deviation is 111.1 nm. Size distribution and Polydispersity Index (PDI) of the smHSSs were also measured by a Zetasizer Nano-ZS (Malvern Instrument). As shown in Fig. 2E, the average size is 843.3 nm. According to the measurement, the PDI is 0.039, demonstrating a good monodispersity of the nanospheres. The SEM images also show the hollow structures with the broken parts and voids. The TEM images further confirm the hollow silica spheres with mesoporous morphology. The thickness of the shell layer is estimated to be ~20 nm.

### Investigation into the synthesis mechanism of smHSSs

To explore the formation mechanism of smHSSs, we conducted a series of experiments to investigate the specific roles of CTAB, NH_3_·H_2_O, flow rate and the length of the spiral microchannels. As shown in Table S1, without CTAB, no obvious hollow structures were observed. Therefore, the existence of stabilizer (CTAB) is required for the synthesis of smHSSs as it determines the formation of shell during the process. Ammonium hydroxide as a catalyst determines the efficiency of the hydrolysis and thus the thickness of the shell layer (Fig. S2). At low flow rate of both flows, no obvious hollow structures were observed neither. This is probably caused by non-complete condensation of hydrolyzed TEOS due to non-sufficient mixing of the two reactant flows.

To demonstrate the effects of channel length in the synthesis of smHSSs, we designed spiral microchannels with only two runs. Though smHSSs could still be synthesized, an unstable spherical morphology was observed (Fig. S3), proving the stabilization function of longer channels for the condensation of hydrolyzed TEOS. We also compared the mixing performance of the spiral microchannel with channels of different geometries (Fig. S4) including expansion and contraction, circular serpentine and rectangular serpentine. For the first design, Dean flow effects are also introduced because of the expansion and contraction regions. However, relatively small dimensions of the channel result in lower throughput as compared to the spiral microchannel (Table S2). In addition, smHSSs fabricated in this channel were irregular rather than spherical (Fig. S5A and 5B), probably due to the expansion and contraction effects. For the latter two designs with curved channels, the opposing curved segments result in the mutually countered Dean effects. Therefore, appreciable mixing in these channels could not be achieved, which lead to irregular products in these channels (Fig. S5C–F).

**Figure 3:**
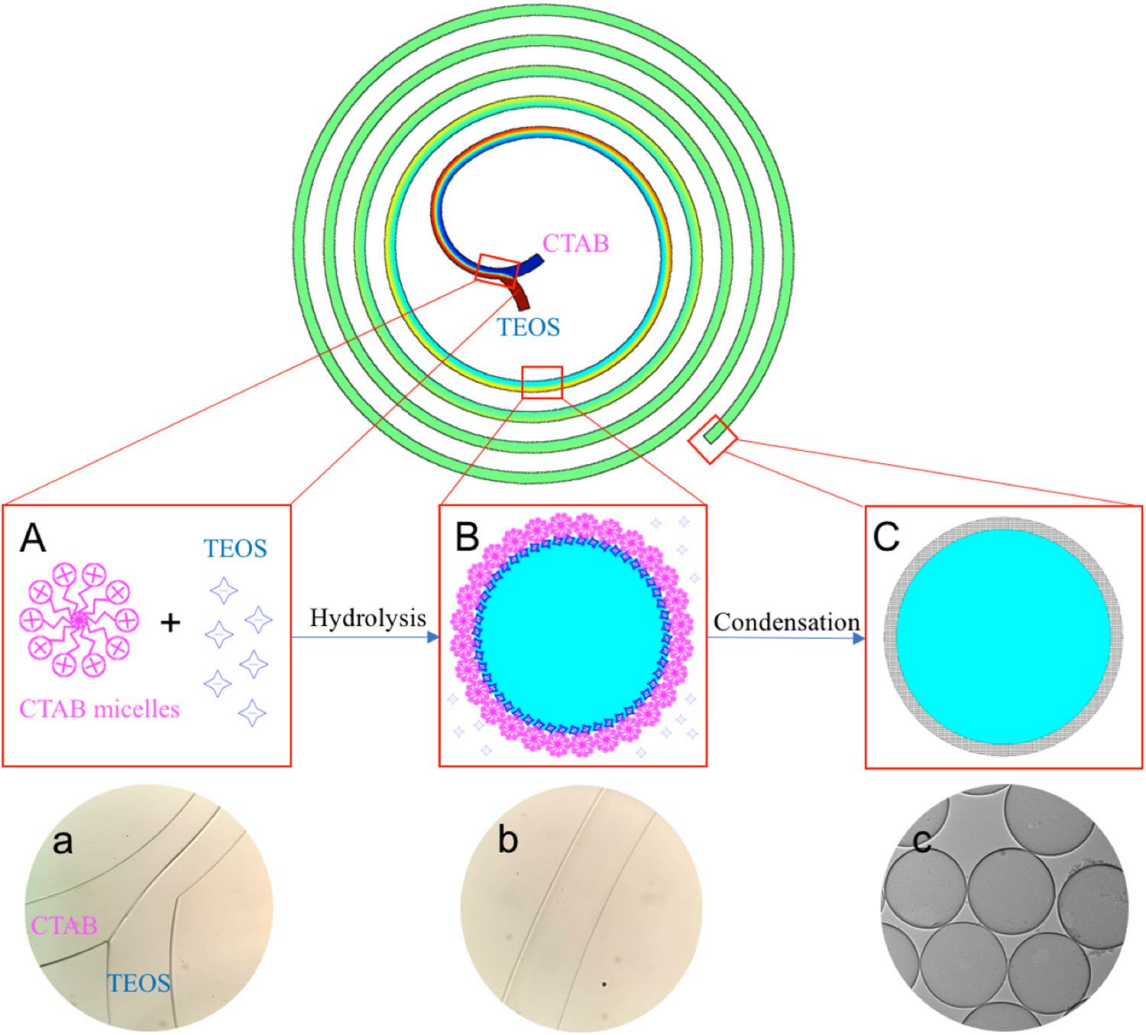
Synthesis mechanism of smHSSs. (**A**) and (a) TEOS in pure ethanol and CTAB micelles in diluted ammonia water were flowed into the spiral microchannel through the inlets; (**B**) and (b) hydrolysis of TEOS occurred at the interface of the two laminar flows near the inlets, then continuous condensation of hydrolyzed TEOS built up the shell layer with a complete mixing of the two fluids; (**C**) and (c) smHSSs were collected at the outlet. (**A**–**C**) show schematic pictures, (a–c) show experimental observations.

Based on the experiments, we proposed a possible formation mechanism for the synthesis of smHSSs in spiral microchannels (Fig. 3). TEOS dispersed in pure ethanol and CTAB spherical micelles formed in diluted ammonia water were flowed into the spiral channel from the two inlets (Fig. 3A and a). At the interface of the two flows, TEOS emulsions were formed and stabilized by CTAB micelles. Then, the fast hydrolysis of TEOS occurred at the existence of catalyst ammonium hydroxide (Fig. 3B and b). With the mixing of the two flows, condensation of hydrolyzed TEOS accumulated to form the hollow spheres (Fig. 3C and c). To further demonstrate the mechanism, we dispersed TEOS in Toluene and then mixed it with CTAB in ammonia water in the spiral microchannel. As Toluene is immiscible with water, the two solutions were nearly not mixed throughout the spiral channel (Fig. S6). No obvious products were observed after the reaction. Therefore, mixing in the spiral microchannel is a key factor for the synthesis.

### Synthesis of multifunctional smHSSs loaded with proteins, fluorescent dyes, quantum dots, and magnetic nanoparticles

**Figure 4:**
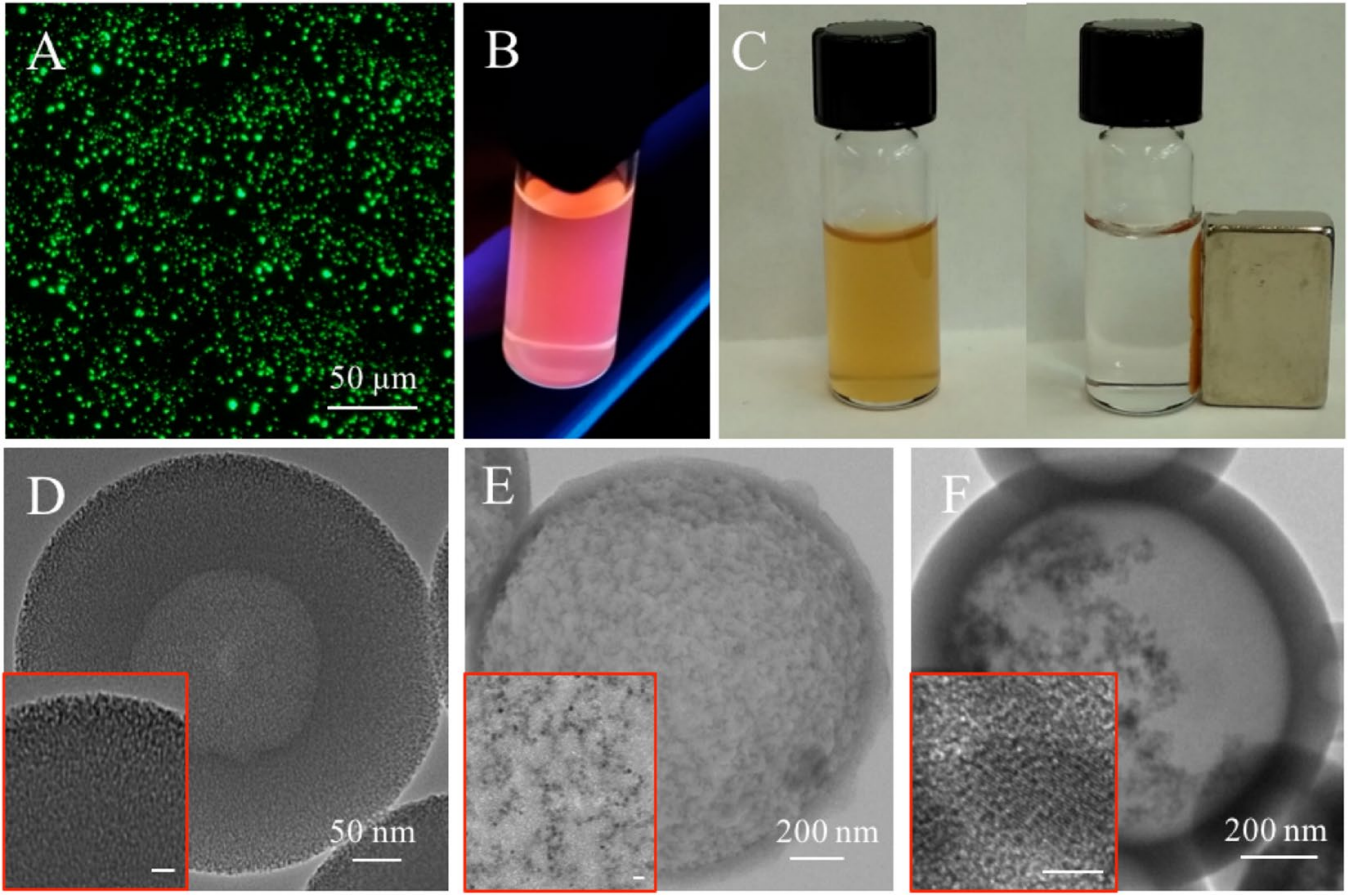
Properties and characterizations of multifunctional smHSSs. Fluorescence images of multifunctional smHSSs loaded with (**A**) BSA-FITC conjugates and (**B**) quantum dots; (**C**) paramagneticity demonstration of multifunctional smHSSs loaded with magnetic nanoparticles; (**D**–**F**) the corresponding TEM images, the insets are high-magnification TEM images, the scale bars for inset images in (**D**) & (**E**) are 20 nm, and for (**F**) is 5 nm.

We further tried to extend the spiral microchannel as a general platform for the synthesis of multifunctional smHSSs integrated with materials such as proteins, fluorescent dyes, quantum dots and magnetic nanoparticles. BSA-FITC conjugates were added into solution of CTAB in diluted ammonia water, and mixed with solution of TEOS in pure ethanol. The final products could display fluorescence signals as shown in Fig. 4A, demonstrating the successful synthesis of fluorescent hollow spheres. The possibility of loading quantum dots (QDs) with smHSSs was proved in similar way. As shown in Fig. 4B, the products were examined with a UV light of 254 nm, demonstrating that the QDs can also be encapsulated into smHSSs. Then, we prepared magnetic nanoparticles (MNPs) and fabricated multifunctional smHSSs loaded with MNPs, with which paramagnetic properties were demonstrated in Fig. 4C. The hollow structures of multifunctional smHSSs loaded with BSA-FITC conjugates were confirmed by TEM as shown in Fig. 4D. To estimate the ratio of BSA-FITC conjugates to smHSSs, we performed thermogravimetric analysis (TGA) of both samples: smHSSs and smHSSs with BSA-FITC conjugates. A weight loss difference of 10.04% (Fig. S7) was measured between the two samples, indicating the successful loading of BSA-FITC conjugates. High magnification TEM images (the inset images in Fig. 4E and F) demonstrate integration of multifunctional nanospheres with QDs and MNPs. We also performed Energy-dispersive X-ray Spectroscopy (EDS) analysis of these multifunctional spheres. The peaks of elements Cd, Se, Zn and S (Fig. S8) further confirm the successful loading of quantum dots and the peaks of element Fe (Fig. S9) demonstrate again the integration of magnetic nanoparticles. According to EDS analysis, the estimated loading amount of QDs and MNPs is 1.6 mg and 11.7 mg per gram silica materials, respectively.

### Applications of multifunctional smHSSs in cell imaging, dye adsorption and drug delivery

**Figure 5:**
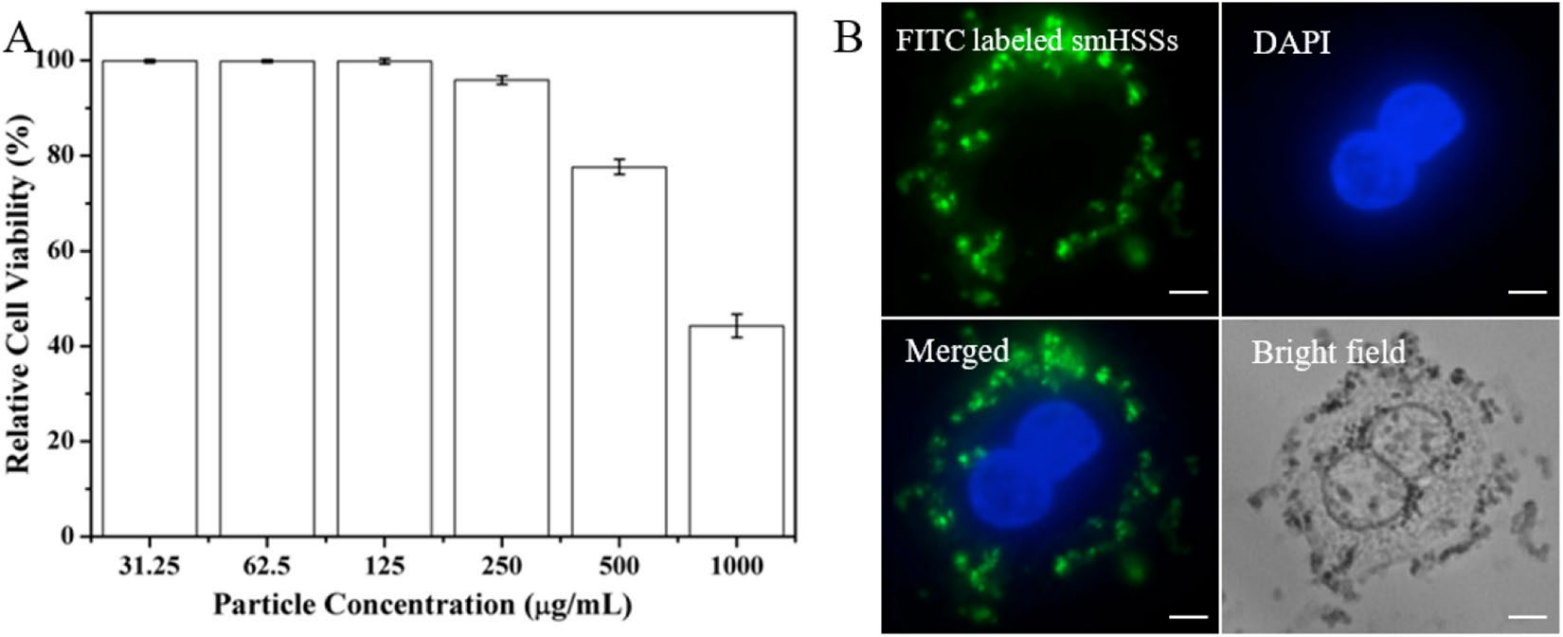
Application of multifunctional smHSSs in cell imaging. (**A**) Cytotoxicity test of the synthesized smHSSs; (**B**) Fluorescent images of SK-BR-3 cells after incubated with FITC-labeled smHSSs for 4 hours, scale bars are 10 µm.

The diversity and functionality of the synthesized multifunctional smHSSs show promising applications in cell imaging, dye adsorption and drug delivery. The cytocompatibility of smHSSs was first examined using CCK-8 method by co-culturing them with SK-BR-3 cells. As shown in Fig. 5A, no significant adverse effects were observed at a particle concentration as high as 500 µg/mL. The cell imaging performance of multifunctional smHSSs was investigated using FITC-labeled smHSSs at a particle concentration of 200 µg/mL. As shown in Fig. 5B, green fluorescence indicates the existence of smHSSs, blue fluorescence from DAPI shows the nucleus of the cells, and the merged fluorescence image demonstrates the uptake of smHSSs by the cancer cells. More obvious evidence could be seen from the comparison of the merged fluorescence image with the bright-field image.

**Figure 6:**
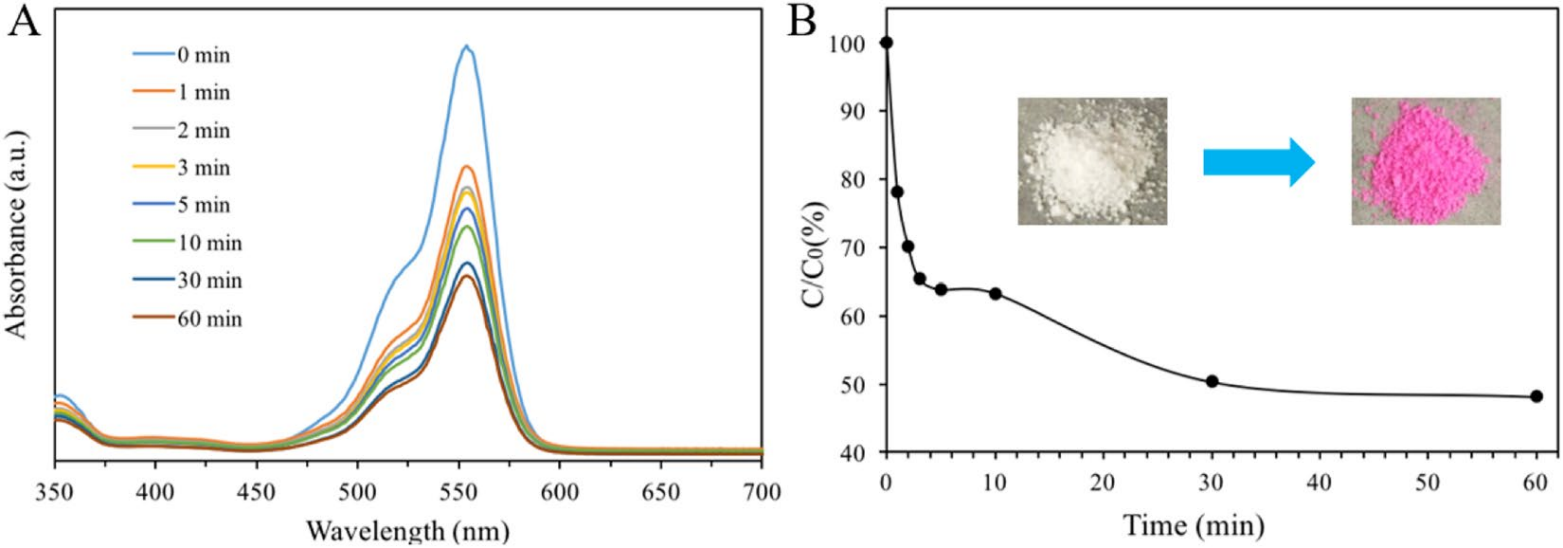
Application of smHSSs in dye adsorption. (**A**) Time-dependent adsorption of Rhodamine B in the presence of smHSSs in dark conditions; (**B**) Adsorption ratio of Rhodamine B (over the initial concentration) by smHSSs. The inset are the images of smHSSs before and after adsorption of Rhodamine B.

Owing to the superior properties of these hollow structures such as large surface-to-volume ratio and porosity, smHSSs may also be applied in fields such as adsorption and separation. To demonstrate this, we examined the adsorption capacity of smHSSs using an organic dye, Rhodamine B. As shown in Fig. 6, at a relatively low mass ratio of adsorbent (smHSSs, 4.50 mg) to adsorbate (Rhodamine B, 0.24 mg)^27–29^, the absorption of Rhodamine B at 554 nm decreased dramatically in the first 5 min, and within half an hour, ~50% of the dye was adsorbed, demonstrating a fast capability and a large capacity of smHSSs towards dye adsorption. The inset picture in Fig. 6B also proved successful Rhodamine B adsorption.

**Figure 7:**
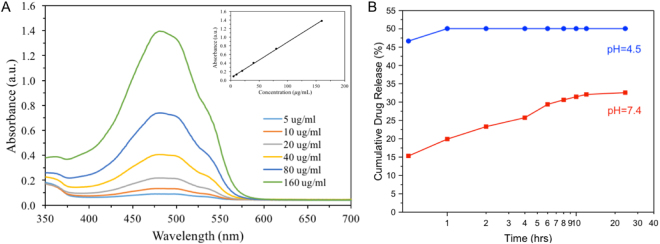
Application of smHSSs in drug delivery. (**A**) Absorbance spectra of different concentrations of Dox in aqueous solution. The inset is the corresponding standard calibration curve of Dox at 488 nm (R^2^ = 0.9996); (**B**) Sustained release profile of Dox from Dox-smHSSs in PBS buffer (pH 4.5 and 7.4). All the values were obtained from the averages of three experiments, and the errors were less than 5%.

Recently, porous silica particles have attracted many interests in biomedicine applications because of their specific roles as drug carriers^30,31^. Drug loading capacity and releasing behavior of smHSSs were explored using doxorubicin (Dox) as a model drug. Firstly, a linear standard calibration curve in the concentration range from 5 to 160 µg/mL (Fig. 7A) was obtained for quantitative determination of the efficiency for drug loading and releasing. Then, the Dox loaded into the smHSSs were measured to be 19.57 mg per gram silica spheres, demonstrating the capability of smHSSs in drug loading. The releasing profiles of Dox from Dox-smHSSs were measured in phosphate-buffered saline (PBS) of pH 4.5 and 7.4. As shown in Fig. 7B, Dox can be successfully released from Dox-smHSSs in both media over 24 hours. This pH-dependent sustained drug release behavior demonstrates attractive application of smHSSs in a controllable way.

## Discussion

We demonstrated a compact platform for the fabrication of uniform-size hollow silica spheres at hundreds of nanometers scale using a flow rate as high as 400 μL/min in a microfluidic spiral channel in less than one second. The geometries of the spiral microchannel were determined by careful considerations towards interface creation and mixing enhancement. Its performance was also compared to microchannels with different geometries. Higher flow rate can further improve the mixing performance of the spiral microchannels as well as increase the throughput. Therefore, the flow rate was determined by examining the formation of smHSSs and calculating the time for synthesis process. Specific roles of CTAB as stabilizer, and ammonium hydroxide as catalyst were investigated for the synthesis mechanism of smHSSs. The feasibility of this platform for producing multifunctional smHSSs was also investigated using proteins, fluorescent dyes, quantum dots, and magnetic nanoparticles. These multifunctional smHSSs could be employed in various fields such as cell imaging, dye adsorption and drug delivery. Many other potential applications could also be explored in the future.

## Methods

### Preparation of chemical solutions

Solution of CTAB in diluted ammonia water was prepared by dispersing 160 mg CTAB in 30 mL DI water (Milli-Q ultrapure water purification system) and then adding 1.5 mL NH_3_·H_2_O. TEOS in pure ethanol was prepared by pipetting 80 μL TEOS into 30 mL 200 Proof Pure Ethanol. The two reactant solutions were then ultrasonically treated until they became transparently clear. All chemicals were used as purchased from Sigma-Aldrich.

### Fabrication of microfluidic spiral channel

The spiral microchannel was fabricated from polydimethryl siloxane (PDMS Sylgard 184, from Dow Corning) using soft lithography. Briefly, film mask was ordered from Fine Line Imaging, Inc. after designing the pattern with CAD. Then the master mold was fabricated from SU-8 3050 using standard photolithography. After that, PDMS replica was obtained by pouring PDMS precursor (ratio of pre-polymer base and curing agent = 10:1) onto the mold and curing the structures at 70 °C for 2 hours. Microchannels were formed by bonding the PDMS replica to a standard glass slide (75 *mm* × 50 *mm*) after oxygen plasma treatment.

### Synthesis of magnetic nanoparticles (MNPs)

Magnetic nanoparticles (MNPs) were synthesized using the method described by Kang *et al*.^32^: water was purified and deoxygenated (by nitrogen gas bubbling for 30 min) and 25 mL of it was mixed with 0.85 mL of 37% HCl; then 5.2 g of FeCl_3_ and 2.0 g FeCl_2_ were successively dissolved in the solution with constant stirring. When seeing an instant black precipitate of Fe_3_O_4_ with the last step, the paramagneticity was checked by placing a strong permanent magnet near the black precipitate. To wash and further purify the MNPs, purified deoxygenated water was added to the precipitate and then it was centrifuged at 4000 rpm for several times.

### Synthesis of multifunctional smHSSs loaded with BSA-FITC conjugates, QDs, and MNPs

The general way is to add different concentrations of target materials such as BSA-FITC conjugates, QDs, and MNPs into the CTAB-ammonia water solution. Taking synthesis of smHSSs loaded with BSA-FITC conjugates for an example, firstly, 5 mg BSA-FITC conjugates was dispersed in 1 mL DI water; after that, 100 µL of the 1 mL solution was added to 5 mL CTAB-ammonia water before mixing with 5 mL TEOS in pure ethanol. For quantum dots (Qdot®625, used as purchased from ThermoFisher Scientific, average diameter 10~15 nm, with a CdSe core and a ZnS shell, emission wavelength = 625 nm), 10 μL (4 μM solution) was used for the synthesis. Magnetic nanoparticles were fabricated by ourselves and the concentration used was 3 mg/mL.

### Cytotoxicity test of smHSSs

Various concentrations (1000, 500, 250, 125, 62.5, and 31.25 μg/mL) of smHSSs were prepared with four repeating tests for each concentration (24 tests). Control and blank groups with four repeating tests for each group were also set up. Firstly, ~5000 SK-BR-3 cells were counted and added into a 96-well plate. The 96-well plate was then placed in an incubator (37 °C, 5% CO_2_) for 24 hours. After incubation, 100 μL smHSSs with different concentrations (1000, 500, 250, 125, 62.5, and 31.25 μg/mL) were pipetted into the experimental groups and 100 μL culture medium were added to each control and blank group. Then the 96-well plate was placed into the incubator for another 24 hours. After that, 10 μL cell counting kit-8 (Sigma-Aldrich) was added to the 30 tests wells. After 4 hour incubation, the absorbance at a wavelength of 450 nm was measured with a plate reader. The survival rate of the SK-BR-3 cells was calculated as [absorbance of (experimental − blank)]/[absorbance of (control − blank)]*100%.

### Multifunctional smHSSs for cell imaging

We demonstrated the biological applications of the synthesized multifunctional smHSSs by performing cell imaging. Thin glass coverslips were sterilized by 70% ethanol before putting into the wells of the 6-well plate. A concentration of ~$1\times {10}^{6}$ SK-BR-3 cells was added into each well and the cells were incubated at 37 °C, 5% CO_2_ for 24 hours. After that, 200 μg/mL of FITC-labeled smHSSs were added into each well and the cell and smHSSs were co-cultured for another 4 hours. Then, the glass coverslips were taken out and observed under the Olympus BX-51 fluorescent microscope.

### smHSSs for Rhodamine B Adsorption

The adsorption performance of smHSSs was evaluated by the adsorptive separation of Rhodamine B in aqueous solution. All the adsorption experiments were conducted under shaking conditions at room temperature in the dark. Typically, the adsorbent (smHSSs, 4.5 mg) was added to Rhodamine B solution (10 mL, concentration 0.024 mg/mL). At appropriate time intervals (0, 1, 2, 3, 5, 10, 30, and 60 min), the aliquots were withdrawn from the suspension and the adsorbents were separated from the suspension via centrifugation of 13000 rpm for 2 min. The concentration of the residual Rhodamine B in the supernatant solution was measured using a plate reader. The adsorption rates were determined at a wavelength range of 350 nm to 700 nm.

### smHSSs for loading and releasing of doxorubicin (Dox)

Firstly, 5 mg Dox (final concentration: 10 mg/mL) was measured and dispersed in 0.5 mL DI water. To load doxorubicin (Dox) into smHSSs, 10 mg smHSSs were weighed and dispersed in the prepared Dox solution. The mixture was under shaking at 37 °C for 48 hours in dark environment. Then, it was centrifuged at 10,000 rpm (for 10  min) and washed twice with DI water to obtain the drug-loaded smHSSs (smHSSs-Dox). The loading amount of Dox was determined by spectra absorbance at 488 nm using a standard calibration curve of Dox. For drug release, smHSSs-Dox sample was immersed in PBS with pH of 4.5 and 7.4, and the supernatant was collected at given time intervals (1, 2, 4, 6, 8, 10, 12, 24 hours). The absorbance spectra at 488 nm was measured to determine the amount of Dox released. Repeating experiments of three times were conducted.

## Electronic supplementary material

Supporting information
