# Supplementary material for: Ultrafast Synthesis of Multifunctional Submicrometer Hollow Silica Spheres in Microfluidic Spiral Channels
Source: Sci Rep. 2017 Oct 3;7:12616. doi: 10.1038/s41598-017-12856-9 (PMC5626769; doi:10.1038/s41598-017-12856-9)
Supplement: Supplementary file 1 — Supporting information [file 41598_2017_12856_MOESM1_ESM.pdf]

Supporting Information

Ultrafast Synthesis of Multifunctional Submicrometer Hollow Silica Spheres in Microfluidic Spiral Channels

Yuan Nie[+], Nanjing Hao[+], and John X. J. Zhang*

Thayer School of Engineering, Dartmouth College, 14 Engineering Drive, Hanover, New Hampshire 03755, United States

*E-mail: john.zhang@dartmouth.edu
[+]These two authors contributed equally in this study.

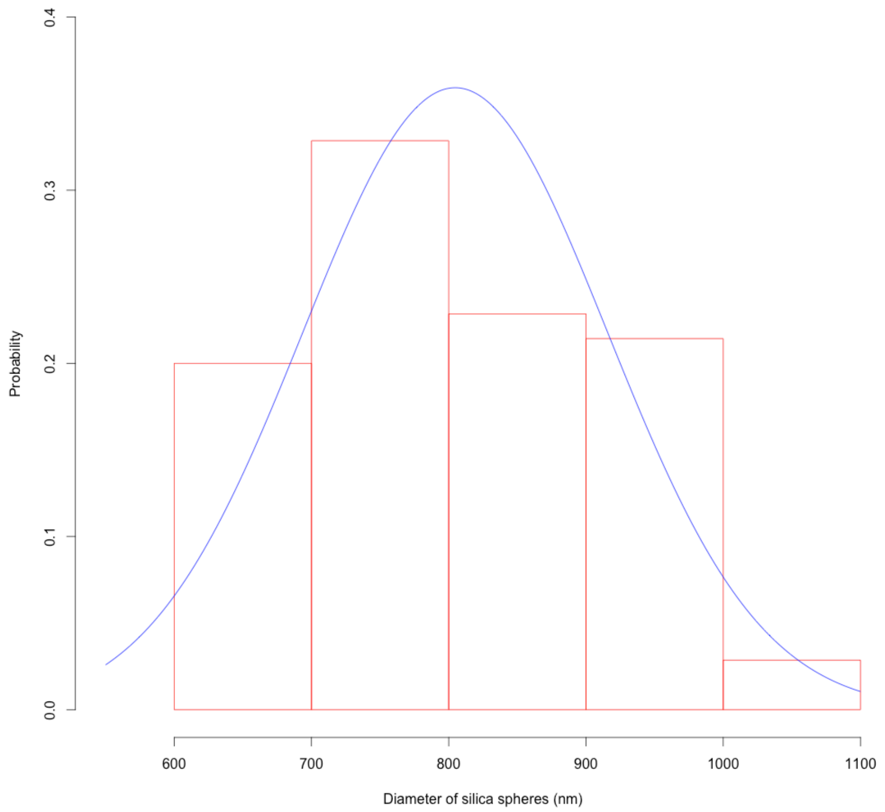

Figure S1. Statistical size distribution of as-synthesized smHSSs, the average diameter of silica spheres is 804.7 nm, and the standard deviation is 111.1 nm (13.8%).

Table S1. Mixing of two reactant solutions with different chemical compositions, concentrations and flow rates for the synthesis of smHSSs. Results for test (1) – (3): no desired smHSSs synthesized; result for test (4): smHSSs synthesized.

Solution II (Inlet 2) / Solution I (Inlet 1)	80 µl TEOS in Ethanol
Diluted ammonia water	(1) 5 µL/min
	(2) 10 µL/min
CTAB in diluted Ammonia water	(3) 5 µL/min
	(4) 10 µL/min

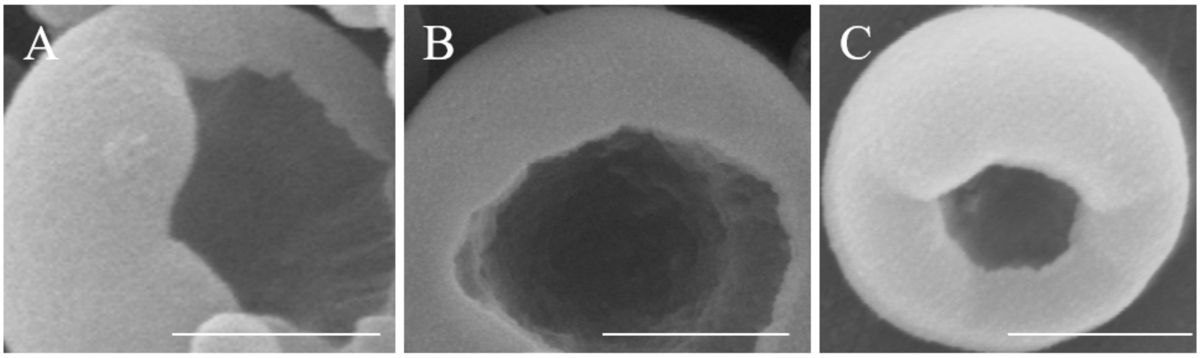

Figure S2. smHSSs synthesized with ammonia water of (A) 0.5 mL, (B)1.5 mL, and (C) 3 mL, scale bars are 500 nm.

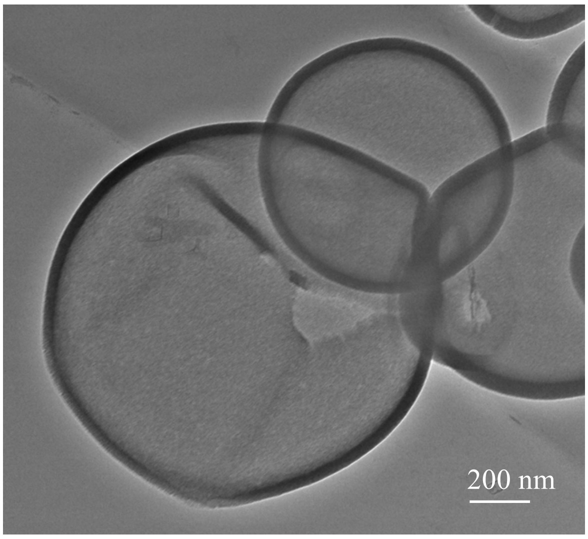

Figure S3. Transmission Electron Microscopy (TEM) image of smHSSs synthesized in two-run spiral microchannel.

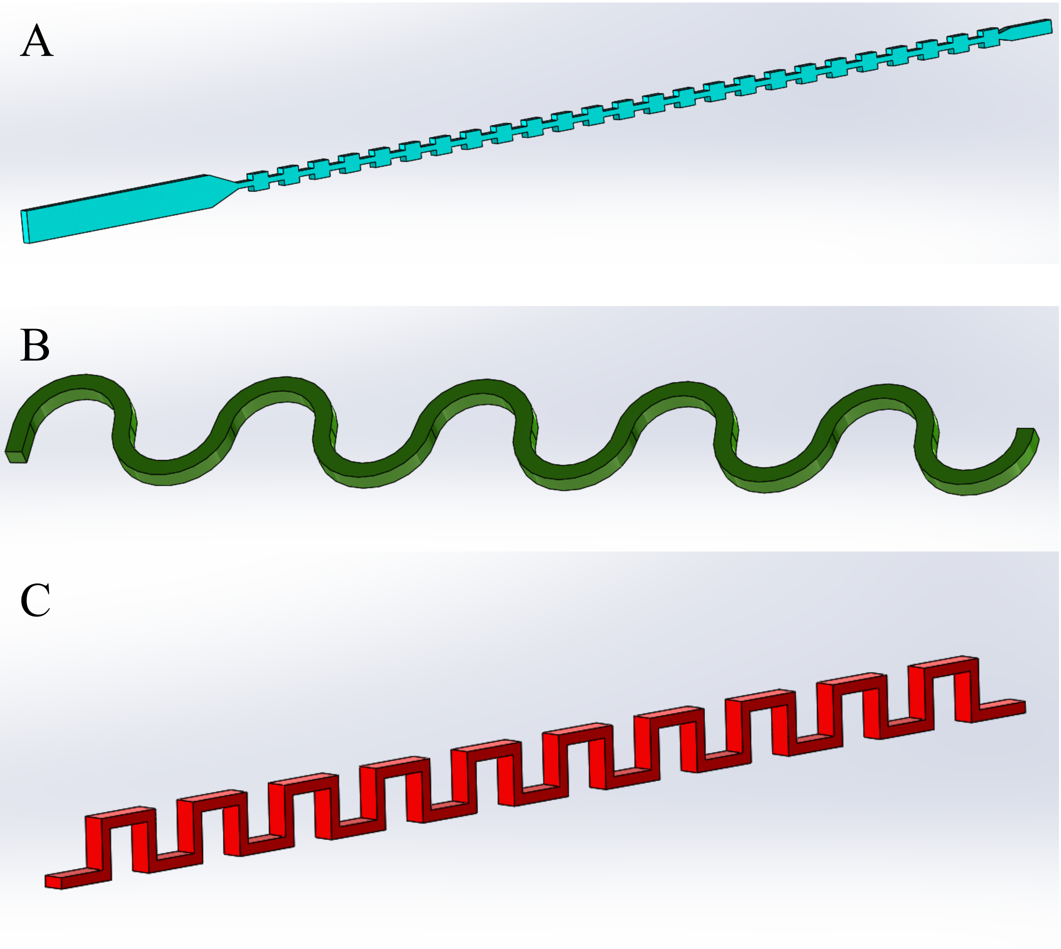

Figure S4. Schematic images of different channel designs: A. expansion and contraction channel; B. circular serpentine channel; and C. rectangular serpentine channel.

Table S2. Setup of synthesis tests with different microfluidic channel designs

<table>
<tr><th>Micro channels</th><th>Channel width (μm)</th><th>Flow rate</th><th>Chemical solutions</th></tr>
<tr><td>Spiral</td><td>500</td><td rowspan="4">400 μL/min</td><td rowspan="4">Solution I: Ammonia water + CTAB

Solution II: 80 μL TEOS in ethanol</td></tr>
<tr><td>Expansion and contraction</td><td>Expansion region: 90
Pinched region: 30</td></tr>
<tr><td>Circular serpentine</td><td>200</td></tr>
<tr><td>Rectangular serpentine</td><td>100</td></tr>
</table>

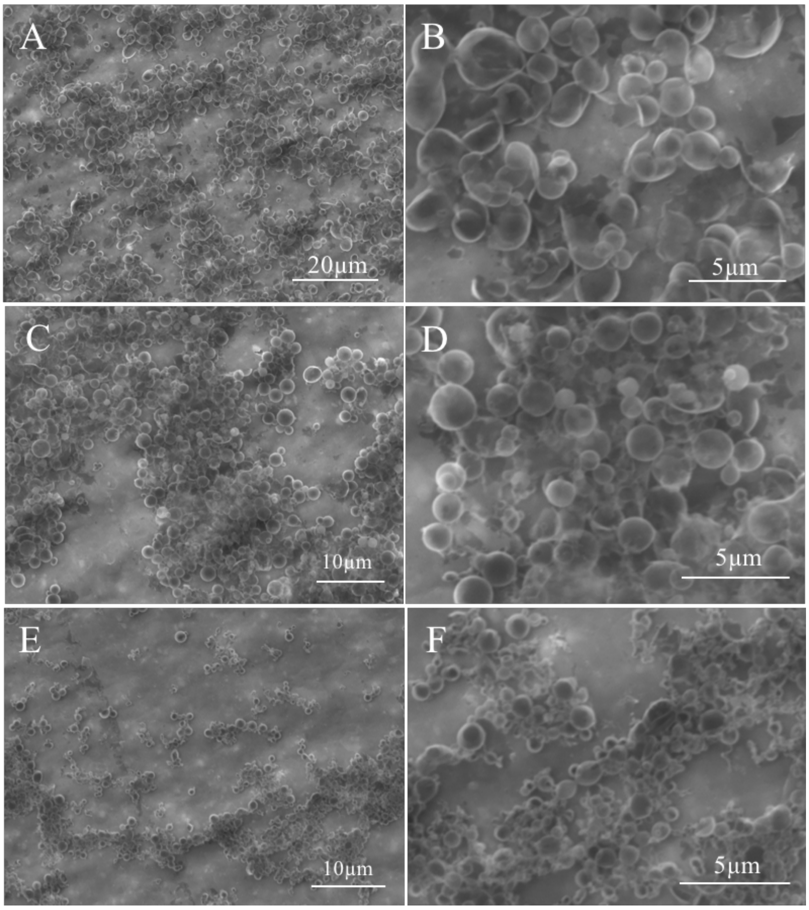

Figure S5. Scanning Electron Microscopy (SEM) images of smHSSs synthesized in expansion and contraction channel (A and B), circular serpentine channel (C and D), and rectangular serpentine channel (E and F).

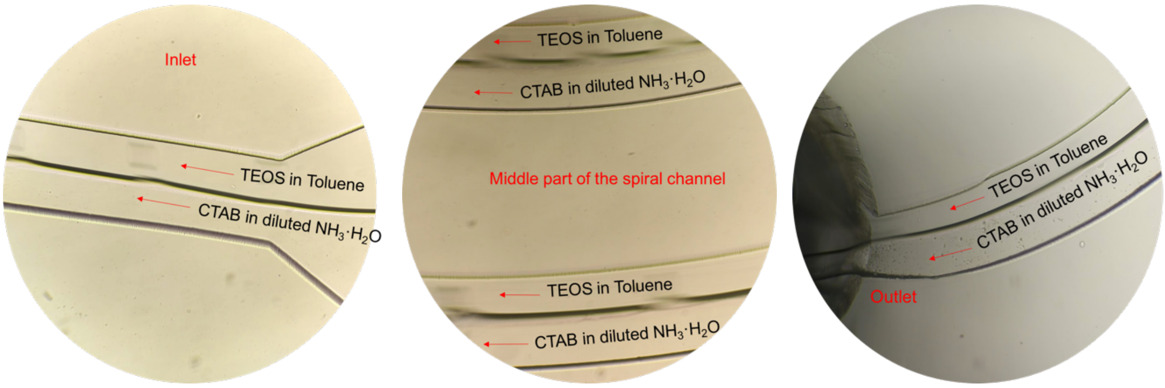

Figure S6. Mixing of TEOS in Toluene and CTAB in diluted ammonia water.

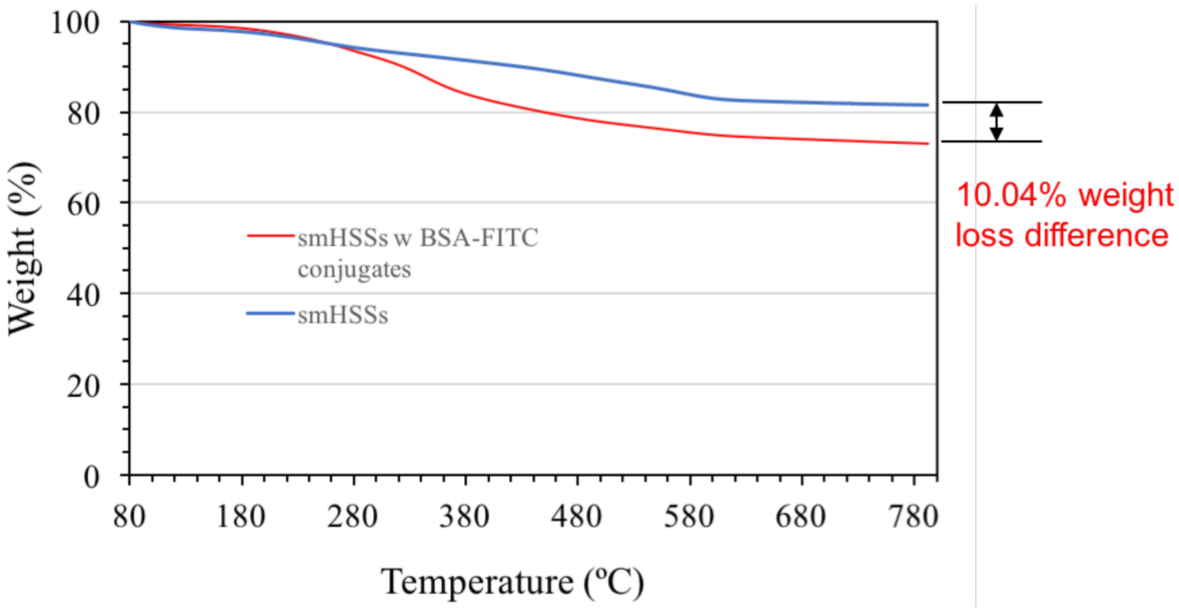

Figure S7. Thermogravimetric analysis (TGA) of smHSSs loaded with BSA-FITC conjugates and smHSSs.

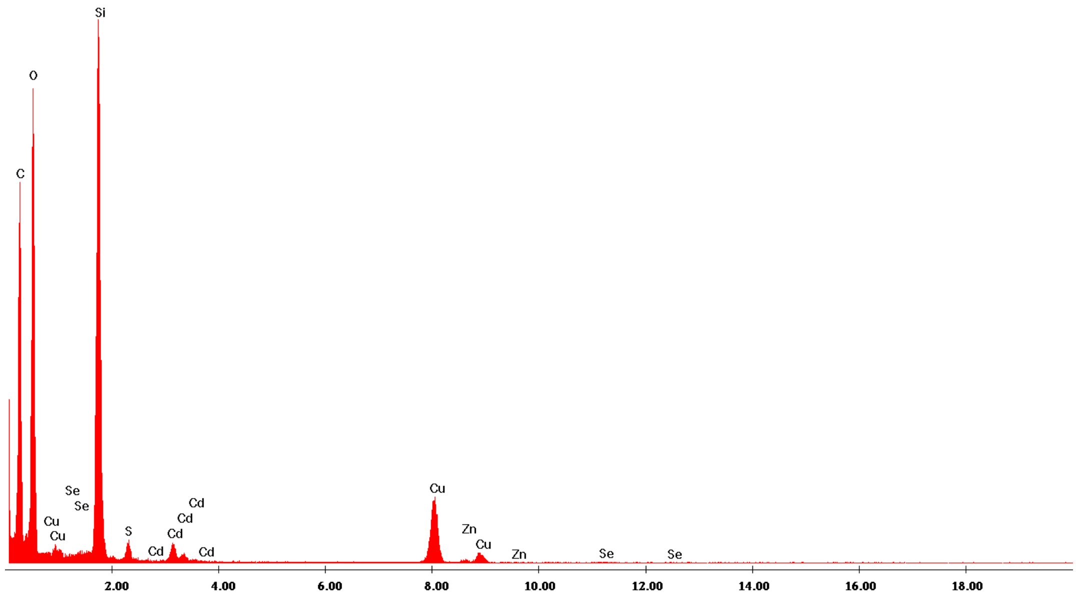

Figure S8. Energy-dispersive X-ray spectroscopy (EDS) analysis of multifunctional smHSSs loaded with quantum dots.

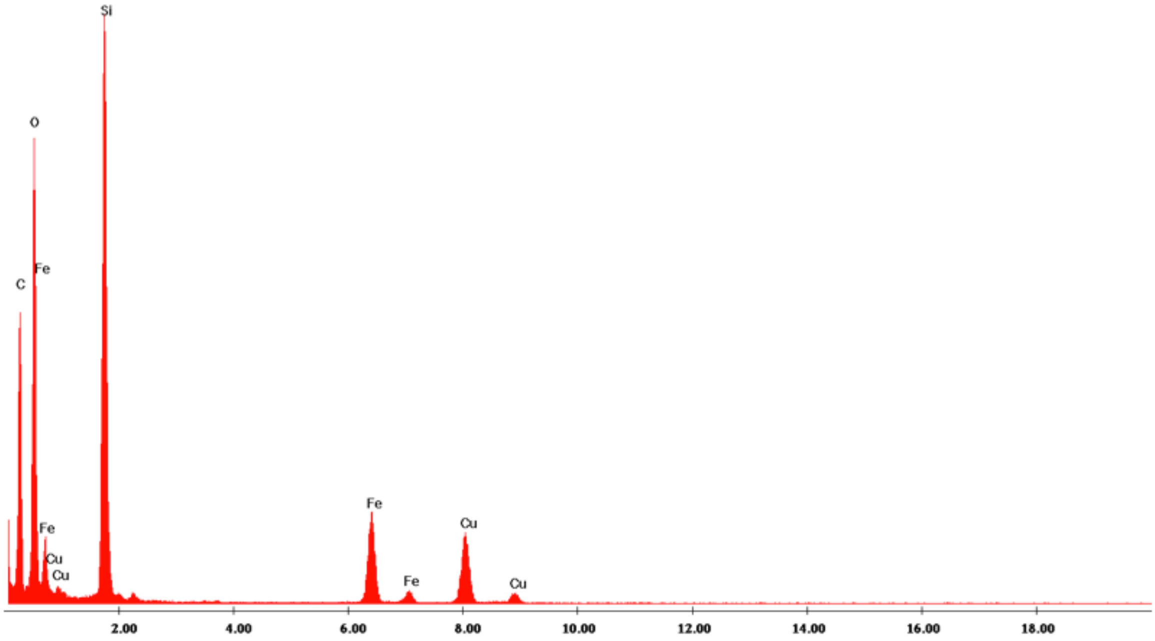

Figure S9. Energy-dispersive X-ray spectroscopy (EDS) analysis of multifunctional smHSSs loaded with magnetic nanoparticles.
